# MTHFR C677T polymorphism and cerebrovascular lesions in elderly patients with CSVD: A correlation analysis

**DOI:** 10.3389/fgene.2022.987519

**Published:** 2022-09-23

**Authors:** Zhuoran Li, Xiaoyan Wu, Haowei Huang, Fan Xu, Guangtie Liang, Chuying Lin, Qinbao Qin, Xiuxia Lei, Xuwen Zeng, Xinqing Jiang, Xinhua Wei

**Affiliations:** ^1^ Department of Radiology, Guangzhou Red Cross Hospital of Jinan University, Guangzhou, Guangdong, China; ^2^ Department of Neurology, The Fourth Affiliated Hospital of Guangzhou Medical University, Guangzhou, Guangdong, China; ^3^ Department of Radiotherapy, Guangzhou Red Cross Hospital of Jinan University, Guangzhou, Guangdong, China; ^4^ Department of Laboratory Medicine, Guangzhou First People's Hospital, School of Medicine, South China University of Technology, Guangzhou, Guangdong, China; ^5^ Department of Laboratory Medicine, The Sixth Affiliated Hospital (Gastrointestinal and Anal Hospital), Sun Yat Sen University, Guangzhou, Guangdong, China; ^6^ Department of Geriatrics Neurology, Guangzhou First People’s Hospital, School of Medicine, South China University of Technology, Guangzhou, Guangdong, China; ^7^ Department of Radiology, Guangzhou First People’s Hospital, Jinan University, Guangzhou, Guangdong, China; ^8^ Department of Radiology, Guangzhou First People’s Hospital, School of Medicine, South China University of Technology, Guangzhou, Guangdong, China

**Keywords:** Hcy, MTHFR gene C677T, senile, cerebral small vessel disease, MRI

## Abstract

Plasma homocysteine (Hcy) has been identified as a potential risk factor for cerebral small vessel disease. Cerebral small vessel disease (CSVD) leads to cognitive impairment, depression, and other symptoms and is a common disease in middle-aged and elderly people. To investigate the relationship between 5,10-methylenetetrahydrofolate reductase (*MTHFR*) C677T polymorphism and CSVD in elderly patients, plasma levels of homocysteine (Hcy) and *MTHFR* genotyping were assessed. MRI and MRA were performed at the same time to analyze the relationship between different genotypes and cerebrovascular lesions. We showed that Hcy plasma levels in the TT group were significantly higher than those in the CC and CT groups. Moreover, we observed that the severity of white matter lesions was associated with women and positively correlated with age, previous coronary heart disease, luminal infarction, and *MTHFR* polymorphism. The multivariate logistic regression analysis showed that age, TT genotype, and lacunar infarction were independent risk factors for white matter hyperintensity (WMH). Importantly, we showed that there was a significant correlation between Hcy plasma levels and *MTHFR* gene polymorphism, with the TT genotype constituting an independent risk factor for WMH. Therefore, we recommended early detection of *MTHFR* gene polymorphisms with concomitant early intervention concerning risk factors to delay the occurrence of cognitive impairment in CSVD elderly patients.

## Introduction

Cerebral small vessel disease (CSVD) refers to a group of diseases captured by clinical and imaging findings, resulting from pathological processes affecting cerebral arterioles, capillaries, and venules (Author Anonymous, 2013). CSVD presents as a chronic, progressive vascular disease that is asymptomatic and easily overlooked in early stages. The pathogenesis of CSVD remains largely unclear. In addition to traditional cerebrovascular risk factors such as hypertension, diabetes, hyperlipidemia, obesity, smoking, and alcohol abuse, hyperhomocysteinemia (HHcy) has been recognized as a new independent risk factor for CSVD in recent years (Homocysteine Studies Collaboration, 2002). HHCy is characterized by high levels of homocysteine (HCys) and represents a key risk factor for cardiovascular diseases.

Clinical data show that hyperhomocysteinemia increases the risk of CSVD (Iso et al., 2004; Khan et al., 2008), displaying a close correlation with multiple luminal infarction and magnetic resonance imaging (MRI) fusion white matter lesions (Hassan et al., 2004). However, on the therapeutic side, not all patients using folic acid to reduce Hcy levels can attenuate the risk of stroke (The VITATOPS Trial Study Group, 2010; Armitage et al., 2010). Moreover, a second analysis is only pertinent in patients presenting with high blood pressure and relatively low platelets (Huo et al., 2015; Spence and Hachinski, 2018). The effect of Hcy on cerebral vasculature has a heterogenic nature and may be affected by several etiological factors. According to recent studies, an increase in Hcy plasma concentration and a C677T polymorphism in 5,10-methylenetetrahydrofolate reductase (*MTHFR*) gene have been confirmed as risk factors for cerebrovascular diseases. Therefore, investigating the relationship between Hcy plasma levels, its related enzyme *MTHFR* gene polymorphism, and CSVD is of great importance for the prevention and treatment of CSVD. The objective of this study was to examine how a *MTHFR* polymorphism influenced Hcy plasma levels to further explore the correlation between CSVD and its risk factors in elderly patients.

## Materials and methods

### Study population

The study population consisted of 304 patients with CSVD admitted from 2017 to present. There are 175 males and 129 females, with a median age of 79.3 (range from 70.5 to 88.1). Peripheral blood was drawn from each subject after obtaining informed consent. Collection of age, sex, past medical history, and drug use data of enrolled patients was performed for those meeting the following inclusion criteria: 1) over 65 years old; 2) MRI examination revealed the presence of CSVD, such as white matter hyperintensity (WMH) and lacunar infarction; and 3) clinical manifestations of CSVD, such as cognitive dysfunction, depression, and gait abnormalities. Exclusion criteria include: 1) previous history of cerebral infarction and head MRA suggesting macrovascular disease; 2) autoimmune or inflammatory diseases, multiple sclerosis, tumor-induced white matter lesions; 3) chronic renal insufficiency and receptor of kidney transplantation; 4) hyper- and hypothyroidism; 5) impaired liver function or previous liver disease; 6) current or recent use of methotrexate, theophylline, B vitamins, folic acid, and antiepileptic drugs.

### *MTHFR* genotyping

For each subject, 2 ml of peripheral blood exposed to EDTA anticoagulant was collected. Genomic DNA was extracted from peripheral blood leukocytes using the modified phenol chloroform extraction method. The *MTHFR* polymorphism was detected using a microarray hybridization PCR kit for the *MTHFR* (C677T) gene (BSK03051, Shanghai Bio Science and Technology Co., Ltd.). The *MTHFR* (C677T) genotype was divided into: wild CC type, heterozygous mutant CT type, and homozygous mutant TT type.

### Plasma homocysteine (Hcy) levels

For each subject, a fasting vein blood sample (3–5 ml) was taken in the morning, centrifuged at 4,000 rpm/min for 5 min, and detected using an AU5800 automatic biochemical analyzer. The reagent was provided by Kingsell Biotechnology Co., Ltd., Wuhan, China.

### Brain MRI/MRA examination and image interpretation

**FIGURE 1:**
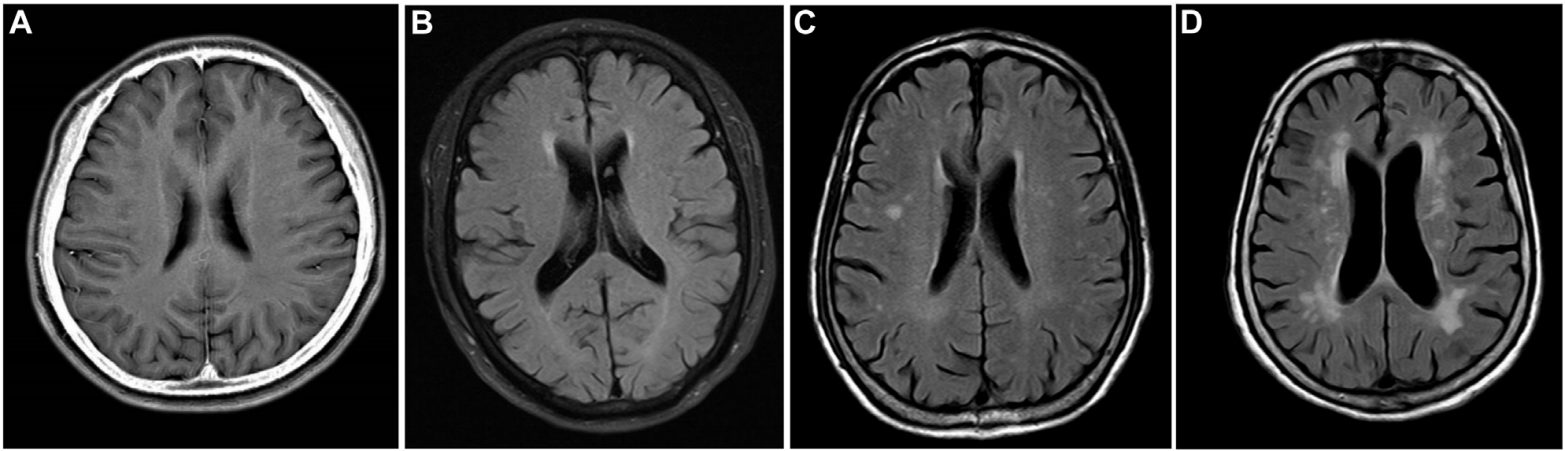
MRI T2 FLAIR image. Grade 0: no white matter lesions **(A)**. Grade 1: focal white matter lesions **(B)**. Grade 2: partial fusion of white matter lesions **(C)**. Grade 3: diffuse involving the entire area **(D)**.

Magnetic resonance imaging (MRI) and magnetic resonance angiography (MRA) were performed using the Philips Achieva 1.5T MRI scanner in accordance with standard operating procedures. All neuroimaging data were evaluated by two independent, accredited, and experienced neuroimaging experts. Depending on the presence of lacunar infarction, two separate groups were set: the lacunar infarction group and the no lacunar infarction group. The degree of white matter damage was classified into four levels according to the age-related white matter change (ARWMC) rating scale, namely, grade 0 = no lesions, grade 1 = focal damage, grade 2 = partial fusion of lesions, and grade 3 = diffuse involving the entire area, with or without “U”-fiber involvement. Depending on the lesion range, two separate groups were set: the absent-to-mild white matter damage group (0–1 grade) and the moderate-to-severe white matter damage group (grade 2–3). Typical images are shown in Figure 1.

### Statistical analysis

Statistical tests of chi-squared (*X* ^*2*^) test, ANOVA analysis, rank sum test, and correlation analysis were used for univariate analysis. Risk factors related to white matter lesions were analyzed by two-class multivariate logistic regression model analysis, in which the stepwise retreat method was used for variable screening. SPSS statistical software (package version 17.0) was used for all the statistical analyses. The test level was *α* = 0.05, and the statistical significance was set at *p* < 0.05.

## Results

### Clinical characteristics

The clinical characteristics are shown in Table 1. A total of 304 patients were enrolled in the present study, including 175 males and 129 females, with an average age of 79.3. There were 127 cases (41.8%) of the CC genotype, 118 cases (38.8%) of the CT genotype, and 59 cases (19.4%) of the TT genotype. Moreover, the study population displayed 217 patients (71.4%) with hypertension, 69 patients (22.7%) with coronary heart disease, and 81 patients (26.6%) with diabetes. No statistical differences were found between the three genotype groups in terms of gender, age, smoking history, previous diabetes history, and hypertension. However, and importantly, in patients with coronary heart disease, the frequency of the TT genotype was significantly higher than that of the CC and CT genotypes (*p* = 0.030). The Hcy plasma levels of the TT genotype were significantly higher than those of the CC and CT genotypes (*p* < 0.001).

**TABLE 1:** Clinical characteristics of patients in three groups of *MTHFR* polymorphism.

	CC (*n* = 127)	CT (*n* = 118)	TT (*n* = 59)	*p*-value
Gender (male/female)	70/57	67/51	38/21	0.479
Age (year)	78.1 ± 8.6	80.0 ± 8.8	80.5 ± 8.9	0.109
Smoking	9 (7.1%)	6 (5.1%)	4 (6.8%)	0.797
Diabetes	33 (26.0%)	32 (27.1%)	16 (27.1%)	0.976
High blood pressure	86 (67.7%)	90 (76.3%)	41 (69.5%)	0.314
Coronary heart disease	20 (15.7%)	30 (25.4%)	19 (32.2%)	0.030*
Hcy (μmol/L)	16.3 ± 5.1	17.5 ± 6.3	20.3 ± 8.2	<0.001*

Data expressed as mean ± S.D. *Statistically significant difference.

### Relationships between *MTHFR* genotype and CSVD

As shown in Table 2, there was a statistically significant difference among the three genotype groups in patients with white matter damage (*p* < 0.001), while no statistical difference was found in patients with lacunar infarction.

**TABLE 2:** Relationships between *MTHFR* genotype and cerebrovascular lesions [n(%)].

Group	Number	Genotype			*X* ^*2*^ */Z*	*p*-value
		CC	CT	TT		
Lacunar infarction	110	45 (35.4)	42 (35.6)	23 (39.0)	*X* ^*2*^ = 0.249	0.883
ARWMC classification						
0	78	45 (35.4)	27 (22.9)	6 (10.2)		
1	66	30 (22.9)	19 (16.1)	17 (28.8)	*Z* = 16.050	0.001*
2	102	36 (10.2)	47 (39.8)	19 (32.2)		
3	58	16 (12.6)	25 (21.2)	17 (28.8)		

*Statistically significant difference.

### Analysis of the relationship between white matter damage and putative risk factors

According to the results of the Spearman correlation analysis shown in Table 3, a weak positive correlation was observed between the severity of white matter lesions and women gender (*p* = 0.010), previous coronary heart disease (*p* = 0.008), luminal infarction (*p* = 0.001), plasma Hcy level (*p* = 0.029), and MTHFR TT genotype (*p* = 0.003).

**TABLE 3:** Spearman correlation analysis of white matter damage.

	R	*p*-value
Gender (female)	0.148	0.010
Age (year)	0.334	<0.001*
Previous coronary heart disease	0.152	0.008*
Hcy (umol/l)	0.015	0.029*
MTHFR TT genotype	0.170	0.003*
Luminal infarction	0.189	0.001*
High blood pressure	0.050	0.385
Coronary heart disease	0.152	0.152

*Statistically significant difference. r, related coefficient.

As shown in Table 4, multivariate logistic regression analysis showed that age (OR 95% CI = 1.042–1.114), TT genotype (OR 95% CI = 1.046–2.279), and lacunar infarction (OR 95% CI = 1.003–3.523) were independent risk factors for WMH.

**TABLE 4:** Multivariate logistic regression analysis of white matter damage.

	B	S.E.	Wald	*p*-value	OR	95% CI
Gender (female)	−0.164	0.290	0.321	0.572	0.848	0.480–1.498
Age (year)	0.075	0.017	19.083	<0.001*	1.078	1.042–1.114
Coronary heart disease	0.348	0.391	0.795	0.373	1.417	0.659–3.048
Lacunar infarction	0.631	0.320	3.883	0.049*	1.880	1.003–3.523
Hcy (μmol/L)	0.022	0.024	0.830	0.362	1.022	0.975–1.070
*MTHFR* genotype	0.434	0.199	4.774	0.029*	1.544	1.046–2.279

*Statistically significant difference. S.E., standard error; OR, odds ratio; Wald = (B/S.E.)^2^.

## Discussion

CSVD refers to diseases caused by small blood vessel lesions in the brain, mainly affecting the arterioles and capillaries of subcortical regions (e.g., basal ganglia and deep white matter). Imaging findings reveal lacunar infarction, WMH, cerebral microbleeds, and perivascular gap expansion in CSVD (Author Anonymous, 2013). Large blood vessel stroke often causes noticeable clinical symptoms, while CVSD is of an insidious nature and can be easily overlooked. The CSVD incidence increases with age, especially in people over 70 years of age, in which CSVD displays an incidence as high as 80% or more (Wahlund et al., 2001; Thal et al., 2012). The relationship between polymorphisms in the *MTHFR* gene and cardiovascular or cerebrovascular diseases is one of the major hotspots in current clinical research. The most common *MTHFR* mutation comprises the C677T point mutation, which may present three polymorphisms (wild CC type, heterozygous mutant CT type, and homozygous mutant TT type). In this study, we investigated the presence of these *MTHFR* C677T polymorphisms in elderly CSVD patients. Our findings showed that there was no statistical difference in gender, age, smoking history, diabetes, and hypertension among the three groups. Importantly, the Hcy plasma levels of TT-type patients were significantly higher than those of CC- and CT-type patients.

In the human body, Hcy is an intermediate product of the methionine cycle. In turn, MTHFR is a key enzyme in the process of folate metabolism, which can catalyze the reduction of 5,10-methylenetetrahydrofolate to n5-methyltetrahydrofolate and transform Hcy into methionine. The *MTHFR* 677 allele C→T mutation causes a decreased MTHFR enzyme activity (Holmes et al., 2011). In fact, Ghodke-Puranik et al. (2015) reported that the enzyme activity of the homozygous mutant TT was 55% lower than that of the CC genotype. Decreased MTHFR enzyme activity may lead to folic acid metabolism disorders, which lead to decreased Hcy methylation, accumulation of Hcy in the body, and, consequently, higher Hcy plasma levels. Accordingly, a recent study showed that for every 5 μmol/L of Hcy in plasma, there was an increased risk of stroke and ischemic heart disease of 59% and 32%, respectively (Moran et al., 2010). Moreover, Wang et al. (2014) reported that the concentration of Hcy required for endothelial damage of the cerebral arterioles was lower than that of the aorta for the same effect, showing a greater sensitivity of small blood vessels in the brain to Hcy. It also confirmed that the *MTHFR* C677T mutation increased the level of Hcy. We now expand this knowledge by showing that Hcy plasma levels of CSVD patients with a TT-type MTHFR polymorphism was higher than those of CC and CT types (*F* = 7.996, *p* < 0.01), which was consistent with the results of domestic and international research.

In this study, the Spearman correlation analysis showed a weak positive correlation between the severity of white matter lesions and MTHFR genotyping, but the logistic regression analysis indicated MTHFR genotyping was an independent risk factor for WMH. The discussion has clarified that there is almost no or very weak positive correlation between the severity of white matter lesions and plasma Hcy levels, and the plasma Hcy level was not an independent risk factor for WMH. The relationship between MTHFR C677T polymorphism and white matter damage is still controversial. Several previous research studies have pointed to Hcy as an independent risk factor for CSVD (Khan et al., 2008; Ma et al., 2010). Indeed, studies have shown that the Hcy plasma levels of patients with white matter lesions are higher than those of patients with other cerebrovascular diseases, and that the degree of white matter lesions increases with higher levels of Hcy. Generally, Hcy can cause lacunar infarction and white matter lesions by damaging endothelial cells, promoting the proliferation of smooth muscle cells, changing the anticoagulant state of the blood system, and participating in the development and progression of atherosclerosis (Wardlaw et al., 2003). The relationship between *MTHFR* C677T polymorphisms and white matter damage is, however, still controversial. A large-scale clinical meta-analysis showed that there was a significant correlation between *MTHFR* C677T polymorphisms (TT and CT types) and the risk of ischemic stroke (Song et al., 2016). In line with this, Rutten-Jacobs et al. (2016) reported that the *MTHFR* C677T polymorphism is closely related to WMH volume. Conversely, Jeon et al. (2014) reported that while Hcy levels are associated with CSVD, there was no correlation between *MTHFR* C677T polymorphism and WMH. Hcy plasma levels are affected by many internal and external factors, including genetics and environment. It is speculated that the effect of *MTHFR* polymorphism on CSVD may be multifaceted, involving the interaction between genes and multivitamins, environment, regions, and races (Lewis et al., 2005). We assumed that drugs taken or other clinical factors might have contributed to the result that there was almost no or a very weak positive correlation between the severity of white matter lesions and plasma Hcy levels. A further prospective study is needed in the future.

In this study, the results of univariate and multivariate logistic regression analyses showed that age is a risk factor for white matter damage, corroborating the unifying hypothesis that age is one of the most important risk factors for WMH (van Dijk et al., 2008). The results of univariate analysis in this study also showed that the severity of white matter damage was associated with lacunar infarction, suggesting that lacunar infarction and white matter damage may interact and promote each other. As age progresses, the denervation of nerve fibers in the white matter arises, and arteriosclerotic vascular changes gradually appear in small cerebral arteries. As the cerebral blood flow gets hypoperfused, the resulting hardened blood vessels cannot maintain white matter blood supply by dilation, and the blood flow of deep perforating arteries is reduced, leading to lacunar infarction, neuronal atrophy, and myelin loss. Therefore, the occurrence and progression of lacunar infarction is closely related to the onset and evolution of white matter damage. Our results confirmed this by showing that the severity of white matter damage is associated with lacunar infarction, suggesting that lacunar infarction and white matter damage may be closely related and interact interdependently.

Altogether, this study showed that Hcy plasma levels are significantly associated with *MTHFR* gene polymorphisms and that the homozygous mutant TT *MTHFR* genotype is an independent risk factor for white matter damage in elderly patients. While clinically it may be insufficient to only focus on Hcy levels, testing for potential *MTHFR* gene polymorphisms in parallel may be advantageous. The TT genotype affects not only folate metabolism but also its absorption rates. As folic acid supplementation only provides a minimal recovery of Hcy levels caused by defects in metabolic enzyme genes, increasing the number of prospective clinical trials is needed to provide a basis for future gene therapy feasibility. Overall, our study recommends that clinicians perform early *MTHFR* gene detection, check Hcy plasma levels, and prescribe folic acid supplementation in time to provide better prevention and treatment of CSVD.

## Data Availability

The raw data supporting the conclusion of this article will be made available by the authors, without undue reservation.
